# Tumor‐infiltrating lymphocyte (TIL) therapy: Historical context, clinical applications, and future directions

**DOI:** 10.1002/cncr.70526

**Published:** 2026-07-21

**Authors:** Shen Li, Alexandra E. Rojek, Reid Shaw, Cristina O’Donoghue, J. Michael Millis, Daniel J. Olson

**Affiliations:** ^1^ Department of Surgery City of Hope Cancer Center Zion Illinois USA; ^2^ Section of Hematology/Oncology Department of Medicine University of Chicago Medical Center Chicago Illinois USA; ^3^ Division of Surgical Oncology Department of Surgery University of Chicago Medical Center Chicago Illinois USA

**Keywords:** adoptive cell therapy, IL‐2, TILs

## Abstract

In the past 15 years, immunotherapy has become a major strategy for cancer therapy. Tumor‐infiltrating lymphocyte (TIL) therapy has been under investigation for nearly four decades and received its first Food and Drug Administration approval in 2024, when lifileucel was approved for patients with previously treated metastatic melanoma. TIL therapy is a form of cell‐based immunotherapy that uses autologous T cells isolated from the tumor microenvironment, which are expanded ex vivo and reinfused to mediate tumor regression after lymphodepleting chemotherapy and high‐dose interleukin‐2. Current data demonstrate durable responses in a subset of heavily pretreated patients with advanced melanoma, and early clinical studies suggest activity in other immunogenic solid tumors. In this review, the historical development of TIL therapy, its current clinical applications, emerging strategies to optimize product composition and the tumor microenvironment, and the critical role of surgeons in TIL procurement and delivery are summarized.

## Abstract 2

Tumor‐infiltrating lymphocyte therapy has reemerged as an effective cellular therapy for advanced melanoma, culminating in the approval of lifileucel with durable responses in heavily pretreated patients. This review summarizes the current clinical evidence, practical considerations for patient selection and toxicity management, and future strategies for improving efficacy and safety of tumor‐infiltrating lymphocyte therapy across solid tumors.

## INTRODUCTION

In the past 15 years, immunotherapy has dramatically improved outcomes by targeting cancers that have escaped endogenous immune surveillance.^1^ The concept of using the immune system to combat cancer was first explored in the works of William B. Coley at the end of the 19th century.^2^ However, not until recent decades have substantial advances been introduced into immunotherapy.

Interferon‐α was the first cytokine‐based immunotherapy approved for cancer, in hairy cell leukemia in 1986.^3^ Shortly after, recombinant interleukin‐2 (IL‐2) was approved for use in metastatic melanoma.^4^ Since the 2010s, immune checkpoint inhibitors (ICIs) that target cytotoxic T‐lymphocyte–associated antigen 4 (CTLA‐4),^5^ programmed cell death 1 (PD‐1) and its ligand programmed death‐ligand 1 (PD‐L1), and lymphocyte activation gene 3^6^ have dramatically improved survival outcomes in select cancers.^7^, ^8^, ^9^ In metastatic melanoma, the combination of PD‐1 and CTLA‐4 therapy has improved median overall survival to approximately 72 months in clinical trials, compared with historical medians of less than 12 months in the pre‐ICI era.^10^

**FIGURE 1:**
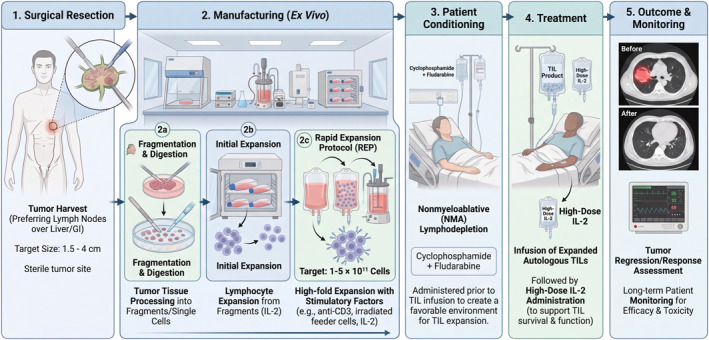
Comparison of adoptive cell therapies over time.

Compared to ICIs, which rely heavily on the body’s in vivo immune system, adoptive cell therapy uses expanded and sometimes engineered lymphocytes to mediate tumor regression.^11^, ^12^, ^13^ The earliest form of adoptive cell therapy, tumor‐infiltrating lymphocyte (TIL) therapy, leveraged preexisting tumor‐reactive intratumoral lymphocytes without the need for T‐cell or antigen selection.^14^ Thereafter, genetically modified techniques were developed, which led to the development of T‐cell receptor, chimeric antigen receptor T (CAR T) cells,^15^, ^16^ as well as neoantigen‐selected TILs (Figure 1).^17^

In 1982, Steven Rosenberg isolated TILs from murine models and demonstrated that combining cyclophosphamide, TILs, and IL‐2 improved disease regression in 50% to 100% of mice with colonic adenocarcinoma with hepatic or pulmonary metastasis.^18^ In 1988, Rosenberg et al. treated 20 patients with metastatic melanoma with TIL therapy and demonstrated a 60% objective regression of disease.^19^ TIL therapy has subsequently demonstrated efficacy against metastatic melanoma, cervical, breast, lung, and colon and rectal cancers.^20^ TIL therapy has been shown to be sufficiently efficacious against refractory metastatic melanoma to warrant the accelerated approval of lifileucel by the Food and Drug Administration (FDA) in early 2024.^21^ In this review, we will discuss the historical perspectives of TIL therapy, current clinical use of TILs cell therapies, next‐generation TIL therapy, as well as the role of surgeons in TIL therapies.

### Historical context of TIL therapy

Early TIL therapy research was limited because of challenges in culturing human lymphocytes ex vivo until the discovery of T‐cell growth factor, IL‐2.^22^, ^23^, ^24^ In 1980, Rosenberg et al. first described lymphokine‐activated killer (LAK) cells. LAKs are a heterogeneous group of cells that consist primarily of natural killer as well as natural killer T and T cells. These cells are generated in vitro by culturing peripheral blood mononuclear cells in the presence of IL‐2.^25^ LAK cells were capable of lysing tumor cells when delivered concurrently with IL‐2.^26^ Objective tumor regression was demonstrated when LAK cells were administered with IL‐2.^27^ However, large numbers of LAK cells were required to demonstrate tumor regression (10^8^ in murine models and 10^10^ in humans). Unfortunately, the high doses of concomitant IL‐2 (approximately 100,000 units per kilogram every 8 hours) drove high rates of toxicity.^28^, ^29^

Rosenberg et al. established a TIL cell population that was 50 to 100 times more potent than LAK cells when used in murine models of sarcoma and colonic adenocarcinoma. In this study, adoptive transfer of 4 to 5 ×10^6^ TILs in conjunction with IL‐2 eliminated 96% of the animal’s pulmonary metastases. The same number of LAK cells had virtually no therapeutic effects. The addition of nonmyeloablative (NMA) chemotherapy, cyclophosphamide (Cy) 100 mg/kg with IL‐2 and TILs resulted in 100% cure of liver metastases. The addition of Cy functioned to deplete regulatory T cells (Tregs) that would normally suppress the antitumor activity of TILs.^18^

**FIGURE 2:**
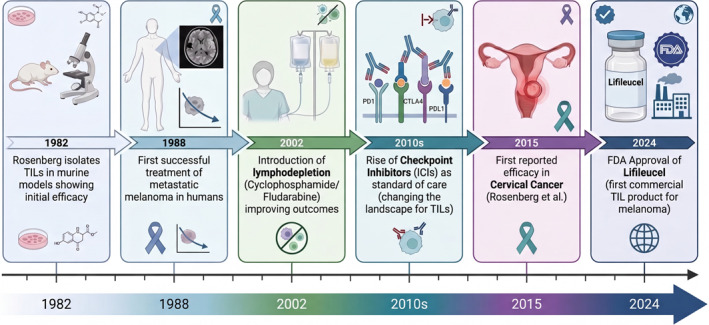
Tumor‐infiltrating lymphocyte (TIL) manufacturing and treatment steps.

Rosenberg et al. then studied the effects of autologous TILs plus IL‐2 in patients with metastatic melanoma. This process consisted of excising tumors into 3‐5mm fragments that were then digested to establish TIL cell cultures. After reaching 1‐5x10^11^, TILs were administered back to patients with IL‐2 in conjunction with or without Cy at 25mg/kg (Figure 2). The overall study demonstrated a response rate of 34% and was similar with or without cyclophosphamide.^30^ The lack of efficacy of Cy in this study could be due to a lower dose compared to the prior animal studies.

To determine whether prior NMA might improve the persistence and function of TILs, Dudley et al. demonstrated that the addition of Cy 60 mg/kg/day for two days and fludarabine 25 mg/m^2^/day (Flu) for five days in patients with metastatic melanoma resulted in objective response in 6 out of 13 patients.^31^ A recent study comparing high‐dose Cy/Flu (120 mg/kg Cy and 125 mg/m^2^ Flu) versus 60 mg/kg Cy and 125 mg/m^2^ Flu demonstrated similarly effective lymphodepletion, although the higher Cy dose led to significantly more adverse effects. Cy doses lower than 60 mg/kg did not achieve adequate bone marrow suppression.^32^ These studies established the key role of conditioning chemotherapy in promoting TIL efficacy and that higher doses did not increase efficacy; however, reduced intensity conditioning regimens, such as those used for CAR‐T cell therapies, have not yet been systematically evaluated in large prospective TIL trials. Emerging retrospective data suggests that reduced‐intensity lymphodepletion regimens (Cy 30 mg/kg/day for two days and Flu 25 mg/m2/day for five days) may be feasible in high‐risk patients, potentially mitigating toxicity while preserving efficacy, although prospective validation is needed.^33^

### Clinical applications of TIL therapy

#### Metastatic melanoma

**FIGURE 3:**
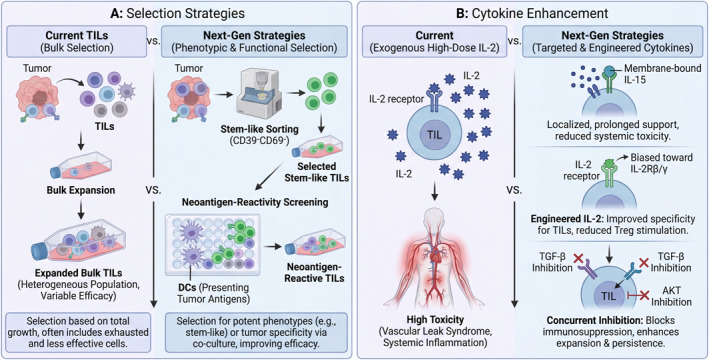
Key immunotherapy milestones before lifileucel approval.

PD‐1 blockade with nivolumab or pembrolizumab alone or in combination with CTLA‐4 or lymphocyte activation gene 3 antibodies is the standard of care for frontline treatment of metastatic melanoma.^34^, ^35^ Combination immunotherapy with nivolumab and anti‐CTLA‐4 has demonstrated a higher response rate, despite a higher incidence of adverse events.^36^ Approximately 50% of patients with melanomas harbor mutations in BRAF, allowing for the use of combined BRAF and MEK inhibition.^37^ Although these therapies offer high rates of disease control, most patients will subsequently progress after BRAF/MEK inhibition. Overall, 50% of patients with stage IV disease will die within 5 years despite these advances.^38^ Begore the FDA approval of lifileucel, there were limited treatment options in patients with metastatic melanoma who have progressed on ICIs or BRAF/MEK inhibitors (Figure 3).

In parallel with ICIs and targeted therapies, TIL research has continued for nearly four decades with objective response rates (ORRs) of 36% to 70% and durable complete responses up to 20%, largely in treatment‐naïve cohorts.^39^, ^40^, ^41^ Despite decades of viability, lifileucel was not approved until February 2024, largely because manufacturing is complex, expensive, and difficult to scale beyond select centers. Costs reflect specialized facilities, labor, and quality control for personalized cell products, and broad access required biopharmaceutical partnership and centralized manufacturing to support a registrational trial.^42^

In the initial cohort of 66 patients in the C‐144‐01 study, lifileucel achieved an investigator‐assessed ORR of 36% (2 complete and 22 partial responses); median duration of response was not reached after a median follow‐up of 18.7 months (Table 1). In a subsequent 5‐year analysis of cohorts 2 and 4, the independent review committee–assessed median duration of response was 36.5 months and median overall survival was 13.9 months, with responses across PD‐L1 status, BRAF status, and prior anti–CTLA‐4 therapy. Toxicities were consistent with lymphodepleting chemotherapy and high‐dose IL‐2, without new long‐term safety signals. Median time to best response was 1.4 months (range, 1.3–8.7).^43^, ^52^

**TABLE 1:** Summary of key clinical trials and efficacy of TIL therapy by tumor type.

Cancer type	Study/Trial	Patient population	Treatment regimen	Key clinical outcomes
Metastatic melanoma	C‐144‐01 (Lifileucel) (Sarnaik et al., Medina et al.)^43^, ^44^	Heavily pretreated; progressed on ICIs and BRAF/MEK inhibitors (if mutated).	NMA Lymphodepletion + lifileucel + high‐dose IL‐2	ORR: 36% (cohort 2) mDOR: 36.5 mo mOS: 13.9 months Led to FDA approval (2024)
	Phase 3 RCT (Rohaan et al.)^45^	Unresectable stage IIIC/IV; 86% refractory to prior anti–PD‐1.	Arm A: TIL therapy Arm B: Ipilimumab (3 mg/kg)	TIL Group: mPFS 7.2 mo; mOS 25.8 mo; ORR 50% Ipi group: mPFS 3.1 mo; mOS 18.9 mo; ORR 20%
Cervical cancer	Rosenberg et al. (2015)^46^	Refractory metastatic cervical cancer.	TIL therapy (HPV‐targeted)	ORR: 33% (3/9 patients) Includes 2 complete responses.
	Phase II (NCT01585428) (Stevanovic et al.)^47^	HPV‐positive cervical cancer.	TILs reactive to HPV E6 and E7 proteins.	ORR: 28% (5/18 patients) 3 partial responses, 2 complete responses.
	Phase II (NCT03108495) (Jazaeri et al.)^48^	Recurrent, metastatic, or persistent disease.	LN‐145 (commercial TIL product)	ORR: 44% (*N* = 27) Disease control rate: 89% at 3.5 mo.
NSCLC	Adjuvant trial (1996) (Ratto et al.)^49^	Postoperative stage II, IIIA, or IIIB.	Arm A: Standard chemoradiotherapy Arm B: TILs + IL‐2	Stage III: mOS 22.4 mo (TIL) vs 8.9 mo (control). Stage II: No significant survival benefit observed.
	Phase I (NCT03215810) (Creelan et al.)^50^	Advanced NSCLC; progression on nivolumab monotherapy.	TIL therapy + nivolumab maintenance	ORR: 23% (3/13 evaluable patients) Median tumor burden reduction of 35%; 2 complete responses.
	Phase II multicenter (Schoenfeld et al.)^51^	Advanced NSCLC; average 2 prior lines of immunotherapy.	Lifileucel (LN‐145)	ORR: 21.4%

Abbreviations: HPV, human papillomavirus; ICI, immune checkpoint inhibitor; mDOR, median duration of response; mOS, median overall survival; mPFS, median progression‐free survival; NMA, nonmyeloablative; NSCLC, non‐small cell lung cancer; ORR, objective response rate; RCT, randomized clinical trial.

Despite promising results, TIL therapy had not been directly compared to ICIs in the PD‐1 antibody refractory setting. In a phase 3 clinical trial, Rohaan et al. compared progression‐free survival in patients with unresectable stage IIIC or IV melanoma. Patients were randomized to TIL therapy or ipilimumab. In this cohort of 168 patients, 86% of patients were refractory to one prior anti–PD‐1 treatment (Table 1). Median progression‐free survival was 7.2 months in the TIL cell therapy group versus 3.1 months in the ipilimumab group. Median overall survival was 25.8 months in the TIL group vs. 18.9 months in the ipilimumab group. Compared to lifileucel, this trial demonstrated a higher objective response rate with 50% in the TIL group vs. 20% in the ipilimumab group. Although cross‐trial comparisons must be interpreted cautiously, the Rohaan trial suggests that earlier‐line TIL therapy and/or use of fresh TIL products may be associated with higher response rates than those observed in the heavily pretreated lifileucel population. Independent of these cross‐trial comparisons, this study demonstrated the superior efficacy of TIL therapy in a refractory melanoma population relative to ipilimumab.^44^

#### Cervical cancer

Outcomes for advanced cervical cancer are poor, with a median survival of only 16.8 months.^45^ The pathogenesis of cervical cancer is driven by infections with high‐risk strains of human papillomavirus (HPV).^53^ HPV‐related oncoproteins E6/E7 can allow for immune tolerance and alterations in the tumor microenvironment but also may be recognized by endogenous immune responses, which may drive a larger population of preexisting TILs in the tumor microenvironment.^54^, ^55^ The standard approach for recurrent or metastatic cervical cancer has been platinum‐based chemotherapy.^56^ Unfortunately, response rates to chemotherapy is poor, ranging from 13% to 17% even with the addition of bevacizumab.^57^, ^58^ Recent studies have demonstrated that ICIs can improve survival in metastatic cervical cancer.^59^, ^60^, ^61^ Unfortunately, responses to ICIs depend on expression of PD‐L1 as well as tumor mutational burden.^46^

Like melanoma, TIL cell therapy offers great potential as a treatment modality. The first reported clinical trial of TIL therapy in cervical cancer was published by Rosenberg et al. in 2015, demonstrating the efficacy of TIL therapy in nine patients with refractory metastatic cervical cancer. Two patients had complete response and one patient had partial response.^62^ A phase II trial (NCT01585428) studied 18 HPV‐positive cervical cancer patients receiving TILs with reactivity to E6 and E7. This study demonstrated that three patients had partial response, whereas two had complete response.^46^ Another ongoing phase 2 clinical trial (NCT03108495) assessed TIL therapy (experimental drug LN‐145) and demonstrated that overall response rate was 44% in 27 patients with a disease control rate of 89% at 3.5 months (Table 1).^47^

### Non‐small cell lung cancer

Non‐small cell lung cancer (NSCLC) often has high tumor mutational burden and endogenous immune infiltration, which confer responsiveness to ICIs. Compared to cytotoxic chemotherapy, ICIs have been shown to improve survival across NSCLC.^48^, ^63^, ^64^ Despite the benefits, resistance often occurs with ICIs, and further therapies are needed. As many NSCLCs exhibit high tumor mutational burden and endogenous immune infiltration, TILs can be expanded from a subset of tumors and offer the promise of overcoming diverse resistance mechanisms to ICIs.^65^, ^66^ The first trial assessing TIL therapy was conducted in 1996 in postoperative patients with stage II, IIIA, or IIIB NSCLC. This study randomized patients to standard chemoradiotherapy or TILs plus IL‐2. No survival benefits were seen in stage II patients, but significantly higher survival rate was seen for stage III patients (22.4 months for TILs vs 8.9 months for standard therapy). Patients received on average 0.4 to 7 ×10^10^ TILs, followed by IL‐2. This was the first study to demonstrate the benefits of TIL therapy in the adjuvant setting.^67^

Dense TIL infiltrates are observed in approximately 25% of patients with NSCLC, more frequently in poorly differentiated carcinomas and in tumors with microscopic vascular invasion.^49^ In a single‐arm open‐label phase 1 trial (NCT03215810), TIL therapy was administered with nivolumab in 20 patients with advanced NSCLC following progression on nivolumab monotherapy. The majority of TILs were harvested from tumors within pleural nodules or supraclavicular lymph nodes. TILs were successfully expanded for 95% of patients. Patients received Cy/Flu, followed by TIL infusion with IL‐2, followed by maintenance nivolumab.

Primary outcome was safety, which was met to the prespecified criteria of <17% rate of severe toxicity. Of the 13 evaluable patients, three had confirmed responses and 11 had reductions in tumor burden with a median best change of 35%. Two patients achieved complete response at nearly 1.5 years.^68^ In another phase 2 multicenter study, lifileucel was used in patients with NSCLC who had received on average two lines of prior immunotherapy. The objective response rate was 21.4% (Table 1).^50^

Although emerging data suggest activity of TIL therapy in other tumor types, including gastrointestinal and head and neck cancers, these studies remain early phase and include small patient cohorts. Accordingly, this review focuses on disease sites with the most mature and clinically relevant data.

### Practical considerations for TIL therapy

#### Patient considerations

With the FDA approval of lifileucel and the expansion of patients treated with TILs as standard therapy, focus has also shifted to the surgical methods required to successfully generate a potent TIL product, as well as the patient factors that promote successful treatment. At present, patients being considered for TIL therapy have advanced disease with progression on multiple prior therapies. Patients should be carefully selected based on discussions in a multidisciplinary tumor board. The intensity of TIL therapy and the physiologic stress induced by IL‐2 also require patients to be closely monitored and selected based on baseline comorbidities (Table 2).

**TABLE 2:** Patient eligibility and surgical considerations for TIL therapy.

Category	Criteria/recommendation	Rationale and clinical context
Patient selection and physiologic reserve	Renal function Creatinine clearance >60 mL/min; serum creatinine WNL.	High‐dose IL‐2 can cause renal dysfunction and significant fluid shifts; baseline function must be preserved.
	Cardiac fitness Functional cardiac reserve required. Contraindications: Decompensated heart failure, high‐risk CAD, or poorly controlled hypertension.	IL‐2 administration induces a “sepsis‐like” state with hypotension and compensatory tachycardia, creating significant cardiac stress.
	General performance status Must be able to tolerate hospitalization and “sepsis‐like” syndrome.	Rehabilitation and optimization of comorbidities are recommended pretreatment to reduce hospitalization‐related morbidity.
	Immunosuppression No requirement for immunosuppressive doses of systemic steroids.	Steroids can dampen the efficacy of the infused T‐cell product.
Surgical resection (harvest)	Tumor size Ideal dimension: 1.5–4 cm.	Sufficient volume is required for successful ex vivo expansion; superficial lesions may not yield enough viable tumor.
	Optimal sites 1. Lymph nodes (highest yield) 2. Lung/thoracic metastasectomies	Retrospective data suggests lymph nodes yield the highest number of TILs; lung lesions also expand frequently.
	Sites to avoid/use with caution 1. Liver: Associated with lowest TIL yield. 2. Gastrointestinal: High risk of bacterial/fungal contamination. 3. Spleen: High number of “bystander” immune cells lacking antitumor recognition.	Liver metastases often have poor viability for expansion. GI contamination can ruin the manufacturing process.
	Surgical approach Minimally invasive (VATS, laparoscopic) preferred over open surgery.	Reduces blood loss and pain; ensures faster recovery so the patient is physically ready for TIL infusion.
Logistics and timeline	Manufacturing wait time Patient must remain stable for ∼5 weeks.	TIL production and potency testing take approximately 5 weeks; rapidly progressive disease (especially CNS metastases) may preclude treatment.
	Tissue handling Maintain at 2°C–8°C; do not fix in formalin.	Fresh tissue is required for cell culture. Tissue for TILs must be kept separate from tissue sent for diagnostic pathology.

Abbreviations: CAD, coronary artery disease; CNS, central nervous system; IL‐2, interleukin‐2; TIL, tumor‐infiltrating lymphocyte; VATS, video‐assisted thoracoscopic surgery; WNL, within normal limits.

IL‐2 administration can cause a sepsis‐like syndrome with hypotension, tachycardia, renal dysfunction, and constitutional symptoms; patients with baseline comorbidities should be carefully evaluated and monitored during TIL therapy. Creatinine should be within normal limits with creatinine clearance >60 mL/min. Functional cardiac reserve and baseline fitness are important given fluid shifts and cardiac stress. Patients with high‐risk coronary artery disease, decompensated heart failure, or poorly controlled hypertension are generally poor candidates. Rehabilitation, geriatric assessment, and optimization of cardiovascular comorbidities may improve readiness and reduce hospitalization‐related morbidity. Potential patients must tolerate all aspects of treatment and be able to wait during TIL manufacturing, which normally requires 5 weeks for TIL production and potency testing.^51^, ^69^, ^70^ Patients should have recovered from prior therapy toxicities (including ICIs) and should not require immunosuppressive steroid doses. Unlike ICIs, TIL therapy can produce durable immune responses with relatively few autoimmune toxicities. However, because it is often given after prior ICI exposure, the physiologic stress of treatment may exacerbate preexisting immune‐related toxicities, particularly adrenal insufficiency. Careful monitoring for hypoadrenalism is warranted, with consideration of stress‐dose corticosteroids when clinically indicated.^51^, ^69^ In patients receiving ongoing immunosuppression for prior autoimmune toxicities, the optimal timing of discontinuation before TIL remains undefined and varies across centers. Supraphysiologic corticosteroids are generally avoided before TIL procurement and infusion because of concerns that they may impair TIL expansion and function.^51^ Data on nonsteroidal immunosuppressants, such as TNF‐α blockade and JAK inhibition, are limited; accordingly, routine concurrent use with TIL therapy should be approached cautiously until further studies are available.

Discharge after TIL therapy occurs at the discretion of the inpatient team but generally follows recovery from acute IL‐2–related toxicities and hematologic recovery.^71^ After discharge, patients are typically seen weekly to twice weekly, as some may require intravenous fluids, transfusions, or other supportive care. This follow‐up also allows monitoring for delayed or less common toxicities, including infection and delayed count recovery. During this period, patients are generally advised to remain near the treating center with caregiver support, although these recommendations may be individualized based on the patient’s recovery trajectory.^51^, ^72^

#### Influence of prior therapies

The influence of prior therapies on TIL efficacy is uncertain. One study that looked at 74 patients with metastatic melanoma found that prior exposure to anti–CTLA‐4 alone resulted in overall response of 38% versus 47% in checkpoint‐naïve patients. Median overall survival was 24.6 months in TIL‐treated patients who were anti–CTLA‐4 naïve versus 8.6 months in patients treated with prior anti–CTLA‐4.^73^ Another study demonstrated TIL therapy mediated a melanoma specific survival of 28.5 months in patients naïve to anti–PD‐1 therapy compared to 11.6 months in patients refractory to anti–PD‐1 therapy, suggesting that earlier use of TIL may lead to superior outcomes. Among patients with BRAF V600E/K mutation, prior targeted therapy was associated with decrease in overall survival compared to treatment naïve patients.^74^ Translational studies have also suggested that melanoma refractory to BRAF/MEK inhibition may be more immune‐evasive and could limit the benefit of subsequent TIL.^75^ It is also commonly observed that patients refractory to BRAF/MEKi often develop rapidly progressive disease and CNS metastases, which can preclude benefit from subsequent therapy, including TIL.^76^, ^77^ Despite this, patients with BRAF mutations responded to lifileucel in C‐144‐01, but the impact of prior therapies on efficacy and manufacturing success remains uncertain.^52^

### Surgical considerations

The primary goal of surgery in TIL therapy is to obtain tissue that supports successful manufacturing of a potent TIL product. It is also important to minimize postoperative complications so patients can receive TIL infusion in a timely fashion.^78^ Therefore, selecting a surgical site for TIL harvest that minimizes morbidity is favored. Although certain lesions are superficial, they may not always yield the required amount of tumor for TIL expansion. In these instances, more complex resections are needed from the thorax or abdomen. A minimally invasive approach is favored as it leads to less blood loss, faster recovery, and improved pain control.^79^ Although no prospective trials have demonstrated optimal site of tumor resection for TIL cell therapy, a retrospective study by Zippel et al. demonstrated that lymph nodes yielded the highest number of TILs, whereas the liver metastatic lesions yielded the lowest numbers. Gastrointestinal metastatic lesions have the possibility of contamination with gut bacteria or yeasts.^80^, ^81^ In another study, TILs generated from metastatic lymph nodes and lung lesions grew more frequently than gastrointestinal derived TILs. Although the spleen can be used, there are significant number of bystander immune cells generated which lack antitumor recognition.^82^ Once the tumor has been resected to 1.5 to 4 cm in greatest dimension, it is processed under sterile conditions. It is important to note that any tissue used for pathology review is kept separate from tissue used for TIL manufacturing. The tumor is stored at 2°C to –8°C until arrival of the courier for transport and pick‐up. In an academic center, TIL products are usually manufactured onsite in a local certified good manufacturing practice facility. In a commercial setting, fresh tumor tissue is shipped to a centralized good manufacturing practice facility to initiate TIL manufacturing (Table 2).^51^

### Real‐world challenges

In real‐world practice, several operational factors influence the delivery and effectiveness of TIL therapy. Manufacturing success is not universal; a subset of harvested tumors fails to yield an adequate TIL product because of insufficient expansion, contamination, or failure to meet potency criteria.^83^ In addition, the interval between tumor procurement and infusion typically takes approximately 5 weeks, representing a critical vulnerability, as patients with rapidly progressive disease may clinically deteriorate and become ineligible for treatment during this period.^70^, ^71^ Bridging strategies may further impact T‐cell fitness or downstream efficacy. These real‐world constraints demonstrate the importance of early identification of appropriate candidates, careful surgical planning to ensure adequate tissue procurement, and multidisciplinary coordination to optimize outcomes.

### Future directions of TIL cell therapy

#### T‐cell phenotypes and tumor microenvironment

**FIGURE 4:**
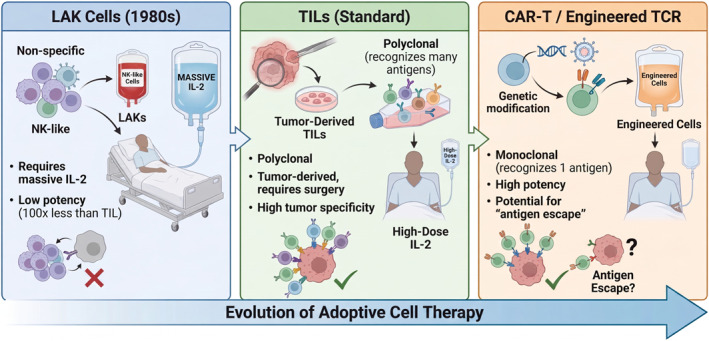
Strategies to enhance future tumor‐infiltrating lymphocyte (TIL) therapy efficacy and safety.

As TIL therapy continues to evolve, the next generation of TILs will ideally improve tumor antigen recognition, leading to more effective tumor responses. Kristensen et al. found that higher frequency and diversity of neoantigen reactive T cells correlated with improved survival in patients with metastatic melanoma compared to patients without T‐cell diversity.^84^ A novel strategy currently in clinical trials (NCT04032847 for NSCLC and NCT03997474 for melanoma) is to isolate TILs and coculture them with autologous dendritic cells that contain peptides corresponding to patient‐specific neoantigens.^85^ This process permits identification and expansion of truly tumor‐specific TILs, which may enhance treatment potency. This strategy underscores the importance of selecting “high‐quality” TILs (Figure 4).

Compared to neoantigen‐reactive T cells, another strategy focuses on using stem‐like TILs. Krishna et al. identified that clinical outcomes in TIL therapy for metastatic melanoma depended on whether T cells exhibited stem‐like versus terminally differentiated phenotypes. Using mass cytometry‐based surface marker analysis, stem‐like CD8^+^ T cells, defined phenotypically as (CD39−CD69−), or double‐negative (DN), resulted in improved clinical response compared with TILs that were (CD39^+^CD69^+^), or double positive.^86^ In further studies of these TIL product populations, DN cells demonstrated a greater proliferative potential and retained tumor recognition, further validated in murine models. CD39^+^ cells are thought to represent chronically stimulated, tumor‐reactive cells, whereas CD69 is a marker for early activation and tissue‐residency, altogether suggesting that this DN population is less chronically stimulated and an earlier progenitor T‐cell state, with higher proliferative potential.

Other studies have also evaluated the role of exhaustion phenotypes in tumor‐specific TILs, and noted that many of these exhaustion profiles initially found in TILs are effectively reprogrammed during expansion with IL‐2 to generate the infusion product.^85^ Specifically, single‐cell RNA and ATAC sequencing in this study revealed that after expansion in IL‐2, chromatin accessibility in key loci of canonical genes expressed in exhausted T cells such as at the *TOX* locus were no longer accessible, and signatures associated with exhaustion scars on chromatin were reversed. However, among responding patients after TIL product infusion, these cells reexpressed exhaustion markers but retained transcriptional signatures of effector function, suggesting that IL‐2 during expansion allows for functional reinvigoration of intermediate cell states with markers of exhaustion that nevertheless retain the plasticity needed for effector function.

The frequency and specificity of neoantigen targeting TILs has also been investigated in TIL efficacy. Early studies identified that a higher tumor mutational burden and predicted neoantigen load were associated with clinical benefit after TIL therapy.^84^, ^87^ More recent explorations of neoantigen‐specificity in TILs have suggested that patients with clinical response had baseline tumors enriched for neoantigen‐reactive TILs that were expanded in the final TIL infusion product and subsequently persisted postinfusion.^88^ Additionally, these tumor‐specific TILs appeared to lose their exhausted transcriptional phenotype after expansion, now displaying an intermediate exhausted effector state. This work suggests that future directions of investigation may benefit from not only identifying and enriching TIL products for neoantigen‐specific cells, but that for a subset of these cells, functional reinvigoration is possible to mediate a clinical response and patient benefit. Ongoing efforts to enhance the specificity of TILs include a strategy to identify neoantigen‐reactive clones during early TIL culture with neoantigen stimulation, resulting in an expanded clonal repertoire of the final TIL product as well as enhanced in vivo antitumor efficacy in xenografted mouse models.^89^

Beyond investigations of TIL phenotypes and neoantigen specificity, the role of the tumor microenvironment and surrounding myeloid cells is an active area of investigation. Transcriptional and cell interaction analyses of melanoma tumors from patients who proceeded to receive TIL therapy revealed that patients who were more likely to respond harbored more interferon‐activated myeloid cell populations, such as M1 macrophages, in addition to CD8^+^ TILs with increased cytotoxicity, exhaustion, and costimulation markers.^90^ The myeloid‐rich environment was reconstituted after lymphodepletion on posttreatment biopsies of responders, and altogether the authors propose that the presence of these myeloid and CD8^+^ TIL interactions enhances antigen recognition, chemotactic interactions, and ultimately antitumor function. This was observed in a population of patients whose tumors had not responded to prior immune checkpoint blockade, suggesting that effective TIL therapy has the potential to overcome this suppressive tumor microenvironment.

Ongoing and future studies are needed to better understand the contribution and interactions of TIL phenotypes, neoantigen specificity, and clonotypic expansion to durable responses after TIL therapy for melanoma. These studies hold promise for future interventions that aim at enriching the TIL product for these desirable attributes. Early studies in murine models have evaluated the role of Akt inhibition in improving persistence of TILs after adoptive transfer.^91^ Other ongoing investigative approaches have targeted transforming growth factor beta (TGF‐beta) receptor on ovarian TILs, resulting in retained strength of cytokine secretion irrespective of presence of TGF‐beta.^92^ Although these interventions need to be further validated in preclinical studies of antitumor efficacy as well as early‐phase clinical trials, they provide early evidence for the feasibility of interventions to improve T‐cell fitness and efficacy in clinical TIL products. Additionally, concurrent therapies which target the tumor microenvironment hold potential to enhance TIL efficacy by leveraging the myeloid component of antitumor immune responses.

#### IL‐2 alternatives and cytokine engineering

IL‐2 is instrumental for T‐cell proliferation during TIL manufacturing and after infusion. The optimal number of post‐infusion IL‐2 doses remains undefined. In the randomized NCI experience reported by Goff et al., IL‐2 was administered to physiologic tolerance rather than to a fixed number of doses, and the number of IL‐2 doses delivered was not associated with response. These data support the commonly adopted toxicity‐adapted approach in which IL‐2 is continued while clinically tolerated and discontinued for significant organ toxicity or physiologic instability, rather than administered to achieve a prespecified dose number.^93^ IL‐2–expanded TILs are more differentiated, leading to early exhaustion and shortened persistence. IL‐2 also has a well‐described high frequency of toxicities.^94^ IL‐15 shares similar structural features to IL‐2 and has been shown to support memory CD8^+^ T‐cell survival without affecting regulatory T cells.^95^, ^96^ In CAR T‐cell production, the supplementation of IL‐15 during manufacturing has been shown to support an earlier memory‐like phenotype, with improved metabolic fitness, and enhanced in vivo antitumor efficacy in murine models.^97^ Early‐phase clinical trials have used this approach with the addition of IL‐15 to culture conditions for anti‐CD19 CAR T‐cell therapy, with improvements in expansion, cytotoxicity, and persistence of these CAR T cells.^98^ This suggests that modifying culture conditions and cytokine exposure for TILs could improve these outcomes, although further study is needed.

Early studies in TILs have explored another strategy to leverage IL‐15 signaling to enhance TIL expansion and persistence through engineering expression of membrane‐bound IL‐15 during TIL expansion. This strategy aims to circumvent the need for IL‐2 administration during expansion under the control of the small molecule acetazolamide.^99^ T‐cell proliferation is supported without the direct toxicity of exogenous IL‐2. A single‐center phase 1 study showed that OBX‐115, an autologous TIL product engineered with regulated membrane‐bound IL‐15, can provide acetazolamide‐controlled cytokine support without postinfusion IL‐2. Early results were promising, with an ORR of 67%.^100^

Toxicities from exogenous IL‐2 exposure not only affect TIL phenotypes and fitness given the use of IL‐2 during expansion of TIL products but also limit the eligibility of patients for TIL therapy who may not be able to tolerate the side effects of exogenous IL‐2 administration. One emerging approach uses an engineered IL‐2 that initiates signaling through the IL‐2 β‐ and γ‐chains, preventing the terminal differentiation of T cells associated with exposure to the α‐chain of IL‐2 (Figure 4).^101^ Further investigation into the potential to apply such principles of IL‐2 structure to TIL expansion both in vitro and after TIL transfer may enhance efficacy and could also expand access and patient eligibility for TIL therapy.

## CONCLUSION

In conclusion, TIL therapy offers substantial promise to become a pillar of cancer immunotherapy across cancer types. However, significant challenges remain. These include poor TIL identification in nonimmunogenic cancers, manufacturing and logistical complexity, and the toxicities of treatment. As demonstrated by the activity of lifileucel in refractory melanoma, TILs now have an established therapeutic role. However, there is considerable room for improving efficacy across all tumor types. Novel technologies improving the isolation and potency of TILs suggest that future TIL therapies may prove more effective and safer than our currently approved therapy.

## AUTHOR CONTRIBUTIONS

**Shen Li:** Conceptualization; writing—original draft; writing—review & editing. **Alexandra E. Rojek:** Conceptualization; writing—original draft; writing—review & editing. **Reid Shaw:** Writing—original draft; writing—review & editing; visualization. **Cristina O’Donoghue:** Conceptualization; writing—review & editing. **J. Michael Millis:** Conceptualization; writing—review & editing. **Daniel J. Olson:** Conceptualization; visualization; writing—original draft; writing—review & editing; project administration; supervision.

## CONFLICT OF INTEREST STATEMENT

D.J.O. reports consulting fees from BMS, Deciphera, Aadi Bioscience, Replimune, Immunocore, Iovance, and Springworks. A.E.R., R.S., C.O., and J.M.M. report no conflicts of interest.
